# Sensitivity and Specificity of the Brentano Illusion Test in the Detection of Visual Hemi-Field Deficits in Patients with Unilateral Spatial Neglect

**DOI:** 10.3390/brainsci13060937

**Published:** 2023-06-09

**Authors:** Maria De Luca, Matteo Baroncini, Alessandro Matano, Concetta Di Lorenzo, Luisa Magnotti, Susanna Lucatello, Martina Mulas, Virginia Pollarini, Maria Paola Ciurli, Davide Nardo

**Affiliations:** 1 IRCCS Fondazione Santa Lucia, 00179 Rome, Italy; a.matano@hsantalucia.it (A.M.); t.dilorenzo@hsantalucia.it (C.D.L.); l.magnotti@hsantalucia.it (L.M.); s.lucatello@hsantalucia.it (S.L.); p.ciurli@hsantalucia.it (M.P.C.); 2 IRCCS Fondazione Stella Maris, 56128 Pisa, Italy; matteo.baroncini@fsm.unipi.it; 3 Faculty of Medicine and Psychology, Sapienza University of Rome, 00185 Rome, Italy; 4 Department of Education, University of Roma Tre, 00185 Rome, Italy; davidenardo@gmail.com

**Keywords:** Brentano Illusion Test, unilateral spatial neglect, specificity, sensitivity, hemianopia, screening

## Abstract

Stroke survivors with right-brain damage (RBD) often present with attentional deficits such as left unilateral spatial neglect. Some patients also present with contralesional visual hemi-field deficits. A late detection of visual hemi-field deficits (VHFD) contributes to hampering neurorehabilitation and functional outcome of patients with neglect. The Brentano Illusion Test (BRIT) may be used for an early detection of VHFD during the neuropsychological assessment. In the present study, we determined the sensitivity and specificity of the BRIT for screening VHFD in patients with neglect. Sixty-four consecutive RBD patients were examined. Forty-five presented with neglect. Of these, 23 presented with VHFD (hemianopia or quadrantanopia) as detected by the Humphrey automated static visual field testing (reference standard). Consecutive patients also included 19 participants without neglect, who did not have any VHFD. The sensitivity and specificity of the BRIT for neglect patients were 78.3% (95% CI: 61.4–95.1) and 90.9 (95% CI: 78.9–100.0), respectively. Positive predictive value (PPV) was 89.6% (95% CI: 76.4–100.0); negative predictive value (NPV) 80.7% (95% CI: 65.2–96.2). No false positives in the group without neglect were identified. We conclude that the BRIT is an effective tool for clinical neuropsychologists to screen for possible VHFD in neglect patients during the neuropsychological assessment, allowing the refinement of the clinical picture in the neuropsychological report. An early detection of VHFD also allows referring the patient to standard diagnostics for a formal visual field examination, right from the first neuropsychological assessment.

## 1. Introduction

Unilateral hemispatial neglect, or hemi-inattention, is a neuropsychological syndrome typically associated with damage to the right hemisphere. The prevalence of the disease after stroke is estimated from 20% to 80%. Such a wide range is determined by many factors, such as time since stroke, lesion site, or screening methodology across studies [1,2]. For instance, a recent study examined a large dataset (about 150,000 consecutive stroke patients), estimating a percentage of 30%, based on the result of a single trichotomic item of the National Institute for Health Stroke Scale [3]. Neglect is more prevalent in patients with right-brain damage (RBD) and affects up to 66% of patients with acute stroke in the right hemisphere [1,4].

Neglect patients show a reduced capability to pay attention to signals coming from the contralesional hemispace, an attentional bias towards the ipsilesional hemispace, and unawareness of objects and events occurring on the contralesional hemispace, despite an integrity of low-level perceptual processing. Thus, they fail to report or respond to stimuli and events occurring on the neglected side. Neglect may affect the visual, as well as other modalities, showing heterogeneous symptoms depending on the spatial frame of reference, portion of space, and processing stage (e.g., perceptual, representational, or motor) involved in the deficit [2].

A double dissociation between visuo-spatial neglect and visual field deficits (VFD) has been demonstrated [5], but neglect can often co-occur with a primary sensory visual loss (unilateral VFD, such as hemianopia or quadrantanopia, or other kinds of visual field loss, e.g., [6]). This may happen, e.g., when cortical lesions (which cause neglect) extend into the occipital regions and/or the underlying subcortical white matter (which cause VFD). The percentage of neglect patients also showing VFD has been reported to range between 34% and 87% [6,7,8,9,10], and may be underestimated in clinical settings because—due to service delivery issues—VFD may remain undiagnosed even after 3–6 months since stroke [11,12]. This yields negative consequences for both the prognosis and neurorehabilitation of neglect patients. Overall, the prevalence of VFD following stroke is estimated between 30% and 70%, with most studies agreeing on a prevalence in stroke of at least 50% [13,14,15,16,17], with hemianopia more frequent than quadrantanopia (65% and 16%, respectively; [16,17]).

The diagnosis of visual neglect relies primarily on paper-and-pencil test batteries based on reading, line bisection, and visual exploration with target-cancelling tasks. Typically, there can be no doubt about the presence of neglect when a patient fails in such tests (e.g., [18,19]). In fact, even if neglect may mask a sensory deficit such as a VFD in the contralesional hemi-field, the opposite does not happen, as patients with only VFD do not fail the tests for the diagnosis of neglect.

A static automated perimetry carried out with the Humphrey Field Analyzer (HFA) is acknowledged as the gold standard in the diagnosis of visual field loss not only for pathologies such as glaucoma, but also in the field of neurology for patients with acquired brain lesions (e.g., [20]). In neurological settings, the integrity of the visual field is conveniently examined with confrontation field testing [20,21], while waiting to carry out a formal perimetry such as the HFA, if there is any suspicion of a VFD. The advantage of confrontation testing is that it can be performed quickly and easily at the bedside, but it requires some expertise as well as choosing the appropriate stimuli and procedure [21], in order to be sensitive. However, an in-depth visual field analysis by perimetry seems to be reserved for selected patients [22], in the sense that, if no suspicion of a VFD exists, a confrontation test is the only assessment of the visual field these patients will obtain. Knowing about the presence of a VFD is very helpful information for refining the clinical picture of a patient in the neuropsychological report. In a neuropsychological setting, the examiner may not have the information about the integrity of the visual field, and may not be sufficiently experienced—or feel sufficiently confident—to perform a reliable confrontation field testing by themselves.

In this context, the paper-and-pencil Brentano Illusion Test (BRIT; [23]) based on the Brentano illusion [24,25], may represent a useful tool for the neuropsychologist assessing neglect patients, since visual illusions can detect a spared visual processing in these patients [23,25,26,27]. The BRIT is a simple test that the examiner can easily administer during the neuropsychological examination (it is a line bisection task). A patient can easily perform the test in about 5 min, without any need to leave their hospital room, or wait for a long time before being referred for a formal perimetry.

The Brentano illusion consists in a single stimulus containing both Mueller-Lyer illusions of line length [24]. That is, one half of the line seems longer (illusion of length expansion), while the other half seems shorter (illusion of length contraction) than they actually are (see Section 2.4, and testing material of [23] available at https://www.tandfonline.com/doi/suppl/10.1080/09602011.2019.1655067 (accessed on 2 June 2023)). The Brentano illusion comes in two configurations, depending on whether the illusion of length expansion is shown on the left or the right side. Together with the Brentano illusions, the test uses also items made up of plain lines (such as the ones used in the standard line bisection task), so that the BRIT outcome relies on both Brentano illusions and neutral configurations (i.e., plain lines).

The rationale behind the use of the Brentano illusion is the asymmetrical sensitivity to the illusion shown by neglect patients with a VHFD [25]. In fact, neglect patients without any VHFD are sensitive to the illusion of length expansion on both sides, whereas neglect patients with a VHFD fail to perceive the illusion on the left side (i.e., asymmetrical effect of the illusion in neglect patients). Therefore, when presented with an illusion of length expansion on the left, neglect patients with a VHFD would bisect to the right of the midpoint, whereas those without a VHFD would bisect to the left of the midpoint [25]. The asymmetrical behavior flags the probable presence of a VFHD (cf. “Rightward Bias” below).

The BRIT has been recently introduced as a formal test that uses the Brentano illusion to detect the presence of unilateral VHFD in neglect patients, reporting normative data on a large sample of healthy individuals [23]. The task requires the patient to perform a line bisection by identifying and marking with a pen stroke the centre of a line printed on an A4 sheet. Based on the bisection of 30 lines varying in length (short and long) and absence/laterality (left and right) of the illusion (i.e., configurations), the BRIT outcome will indicate whether a neglect patient’s performance reveals the presence or absence of a VHFD. An outcome of a “Rightward Bias” in the BRIT index labelled “Symmetry of the Illusory Effect” (SIE; see Section 2.5.2) indicates asymmetrical sensitivity to the illusion, and thus the probable presence of a VHFD. A Rightward Bias in the SIE is not simply a deviation of performance to the right as typically shown by neglect patients, but a deviation that exceeds a given cut-off that takes into account the co-presence of neglect and VHFD. If the BRIT is accurate, the SIE scores in neglect patients with a VHFD should flag a Rightward Bias (i.e., sensitivity of the BRIT); conversely, the SIE scores in neglect patients without any VHFD should flag a “Normal” outcome (i.e., specificity of the BRIT). Therefore, if the BRIT is accurate, it may represent a useful tool for an early detection of a VHFD during the neuropsychological assessment.

The main aim of the present study was to determine the sensitivity and specificity of the BRIT as a screening test for VHFD in patients who, following a stroke in the right hemisphere, developed neglect. To the best of our knowledge, this has never been investigated in previous studies. Indeed, the Brentano illusion has been used only in a few patients (i.e., seven neglect patients with VHFD, seven neglect patients without VHFD, and six patients with hemianopia without neglect [25,26,27], and in three single cases [23] to illustrate examples of application of the BRIT). Therefore, we enrolled a larger sample of consecutive RBD stroke patients routinely referred to assessment for neglect. Our participants included both patients diagnosed with neglect, and patients without neglect. The inclusion of the latter allowed us to explore how the BRIT performs in RBD patients in general, to complement the information available about the healthy population [23].

We also have a number of subsidiary, more practical goals, stemming from our daily experience in the Hospital, that is, to address some questions routinely asked by neuropsychologists about the BRIT and not addressed in the work introducing this test [23].

The first is whether a high intra-individual variability of performance across trials may impact on the sensitivity and specificity of the BRIT. Neglect patients typically make variable responses when bisecting BRIT trials within the same stimulus condition (e.g., they may alternate rightward, central, and leftward bisections with respect to the midpoint). Outlying responses may influence the scores computed in the BRIT, due to the small number of trials per condition. Hence, in addition to presenting our main results—computed using means (as indicated in [23])—we also recomputed sensitivity and specificity using medians (notoriously less sensitive to outlying values), to see whether the latter differed from the main results.

A second question concerns the fact that in different patients the SIE may flag a Rightward Bias either in both line lengths (long and short), or just in one. Therefore, the neuropsychologist may wonder whether one flag is enough, or both are needed to suggest the presence of a VHFD. We could not retrieve any information about this in previous works. In fact, of the three clinical cases reported in the work introducing the BRIT, the only one with a VHFD showed a Rightward Bias in the SIE score flagged in both line lengths [23]. Therefore, we checked for the possible association between the number of lines flagging a Rightward Bias in the SIE score and the accuracy of the test.

Finally, we addressed what to do with and how to interpret the “Length Effect” between the long and the short plain lines (that is, when the long plain line shows a percentage of deviation greater or smaller than the short plain line), and the “Leftward Bias” in the SIE score (that is, when the “Symmetry of the Illusory Effect” exceeds the cut-off on the left side).

## 2. Materials and Methods

### 2.1. Participants

Eighty inpatients with acquired right hemisphere brain damage consecutively admitted to the Neurorehabilitation Unit at Fondazione Santa Lucia in Rome (Italy) were enrolled in this study. The inclusion criteria were: age ≥ 18 years; (ischaemic or haemorrhagic) lesion following a stroke in the right hemisphere; having undergone a standard neuropsychological assessment for the diagnosis of unilateral spatial neglect. The exclusion criteria were: bilateral or left hemisphere lesions (based on neuroimaging data); presence of severe cognitive deficits preventing the comprehension of the tasks; left handedness; previous history of brain lesions, other neurological diseases, or psychiatric diseases/drug abuse. The study was approved by the local Ethics Committee (CE/PROG.839 29-05-20). All patients provided their written informed consent to participate in the study.

The sample size of neglect patients was set for a screening study, considering a prevalence of 50% of VFD in stroke patients with neglect [6,7,8,9,10], with pre-determined null and alternative hypotheses of 50% and 80%, respectively, 0.8 statistical power, and a significance threshold of *p* < 0.05 [28]. The minimum required number of neglect patients was 40 (with 20 as the minimum sample size for a positive case).

Of the eighty participants enrolled, one patient dropped out, and fifteen were excluded for the following reasons: after careful inspection of the neuroimaging data, two patients showed prior brain damage and two patients showed a bilateral stroke; the automated standard perimetry was either not possible or not reliable in eleven patients. Therefore, the final sample included 64 patients. Table 1 reports the demographic and clinical data of all participants, by group. Patients with and without neglect were comparable for all demographic variables. Supplementary Table S1 shows the individual demographic and clinical data of all 64 patients.

### 2.2. Standard Neuropsychological Assessment of Neglect

As routinely assessed after RBD stroke, patients were administered a thorough neuropsychological battery that included standard tests for neglect (e.g., as in [10]), including: Letter cancellation ([29,30]; cut-off: difference ≥ 4 omissions between the left and the right side); Star cancellation ([31,32]; spatial bias cut-off: difference ≥ 3), Line cancellation ([30,33]; spatial bias cut-off: ≥2); Apples Test ([34,35]; cut-offs: ≥6 omissions for accuracy, asymmetry score ≥3 and ≥2 for space centred and object centred neglect, respectively), Line bisection ([36,37]; cut-off > 6.73 mm), Wundt-Jastrow illusion ([38] cut-off: difference ≥ 2 between unexpected responses for left-oriented minus right oriented stimuli), Sentence reading ([30]; cut-off ≥ 1 sentences read incompletely on the left side), Personal neglect ([39]; cut-off: asymmetry score ≥ 2), Room description ([39]; cut-off: ≥1). Patients were classified as having neglect (N+ from here on) if they failed at least two tests (e.g., cf. [37,40,41,42]), or as not having neglect if they did not fail or fail only one test (N–, from here on).

### 2.3. Standard Visual Field Assessment

The Humphrey automated static visual field testing (i.e., the reference standard for the present study) was performed by a chartered orthoptist with a Humphrey Field Analyzer 3 (Zeiss Meditech, Jena, Germany) examining central 24-2 threshold using the SITA Faster strategy with stimulus size III. The test was performed separately for the left and right eye. The VFD were classified as: left hemianopia; left (superior or inferior) quadrantanopia; or absent.

### 2.4. The Brentano Illusion Test (BRIT)

The BRIT [23] is a paper-and-pencil assessment tool based on a Brentano variant of the Mueller-Lyer illusion of length [24] applied to a line bisection task. Patients are asked to cross out the centre of a red horizontal line with a pencil or a thin-tipped marker. The stimuli used for the present study were downloaded from the Supplementary Materials to [23] (from here on: “Original Work”; https://www.tandfonline.com/doi/suppl/10.1080/09602011.2019.1655067 accessed on 5 December 2019). The stimulus set is made up of 30 items (i.e., 5 trials × 6 stimulus conditions; the latter made up of 2 lengths × 3 configurations), separately printed in the centre of an A4 sheet in landscape orientation. The two lengths (80 mm and 160 mm long lines) are presented in three different configurations, two of which contain an optical illusion of length expansion (one on the right and one on the left; i.e., configurations with the Brentano illusion), while one does not contain any illusion (i.e., plain line or neutral configuration). The configurations with the illusion consist of a red horizontal line intersected by three black wings. Within the same line, the “wings-out configuration” forms an angle of 135° with the line and determines an illusion of length expansion of the segment, while the “wings-in configuration” forms an angle of 45° with the line and determines an illusion of length contraction [24]. The neutral configuration is the red line without wings, which is 2 mm thick. The wings are 2 mm thick, formed by two segments 20 mm long for the 80 mm line, and 40 mm long for the 160 mm line.

The BRIT was administered in a single session after the patient gave consent to participate in the study. The BRIT was administered and scored blind to the initial diagnosis, lesion site, and the automated perimetry outcome. The testing procedure, instructions, and scoring followed those described in the Original Work’s package, as did the presentation order of test items (i.e., fixed randomization). Each participant was assessed individually in a well-lit room, comfortably seated at a table in a central position facing the examiner. The sheets were placed (one at a time) centrally with respect to the midline (sagittal plane) of the participant, who was instructed and then introduced to the task by four familiarization items (which were only used as examples, and neither analysed, nor used to compute any score). All participants performed the task with the right hand, and were free to move their eyes and head.

### 2.5. Data Analysis

#### 2.5.1. Group Comparisons

Descriptive statistics for demographic and clinical data, battery for neglect scores, and BRIT scores (cf. Table 1) were computed separately for N+ and N– patients, and N+ patients with (N+H+ from here on) or without VHFD (N+H– from here on), as determined by the standard visual field assessment (see Section 3.2). Group comparisons were computed by running *t*-tests for independent measures (*t*-tests for unequal variances were used in case of significant Levene’s test), or Mann–Whitney tests, and corrected for multiple comparisons using Bonferroni correction (alpha lowered to *p* < 0.00114).

#### 2.5.2. BRIT Scoring and Analysis

Offline, the examiner measured the 30 bisections and entered the deviations one by one in the Excel file named “BRIT Spreadsheet.xlsx” (from now on “Spreadsheet”) provided as Supplementary Materials to the Original Work. BRIT scores were computed strictly sticking to the recommendations and computation rules indicated in the publication (N.B., not in the file “BRIT Scoresheet.pdf”, where cut-offs seem to be inconsistent with both the Spreadsheet and the paper’s content). Each deviation was computed as the distance between the bisection marked by the patient and the midpoint of the line, rounded to the nearest millimeter, using negative and positive values for left and right deviations, respectively. The age of the patient must be entered in a specific cell at the top of the Spreadsheet. The data entered are then automatically processed by the functions embedded into the Spreadsheet (using means to compute values; cf. Section 2.5.4), adjusted for age, and returned as scores in the “Deviation” columns (both in millimeters and as percentages), separately for the 5 indexes of the BRIT. These are: (a) two Line Bisection scores (i.e., LB80 for the short, and LB160 for the long plain line); (b) the Length Effect (LE) score (i.e., the difference between the long and the short plain line bisections); and (c) two Symmetry of the Illusory Effect (SIE) scores (SIE80 for the short, and SIE160 for the long line; ***SIE = [illusion of length expansion on the left − plain line] + [illusion of length expansion on the right − plain line]***); where zero, negative, and positive values indicate symmetry, leftward, and rightward asymmetry, respectively. Based on these scores, the BRIT outcomes are represented by the flags “Normal” or “Bias”, in the adjacent “Result” column in the Spreadsheet. A “Rightward Bias” in the SIE scores is flagged when a deviation to the right exceeds the cut-off values (7.8 mm or 19.5% for the short line, and 11.8 mm or 14.75% for the long line), and indicates the presence of a left VHFD in neglect patients. The flag may show up either in one or both SIE scores (cf. Section 3.4 and Discussion). It is worth noting that deviations to the right are typical of neglect. However, a Rightward Bias in the SIE reflects the presence of a deficit that goes over and beyond the symptomatology of neglect (i.e., the co-presence of a VHFD). The Rightward Bias in the SIE does not depend only on a specific configuration (e.g., the illusion of length expansion on the left) but rather on the algebraic sum of all configurations (e.g., plain lines and illusions of length expansion on the left/right).

#### 2.5.3. Sensitivity and Specificity of the BRIT

The sensitivity, specificity, positive and negative predictive values (PPV and NPV), and related confidence intervals (CIs) were computed based on the classification output (true/false positive/negative cases), determined as follows. A patient was classified as: (a) true positive if the disease was present (i.e., a VHFD ascertained by the reference standard), and a Rightward Bias was flagged in the SIE scores; (b) false positive if the disease was absent, and a Rightward Bias was flagged in the SIE scores; (c) false negative if the disease was present, and a Rightward Bias was not flagged in the SIE scores; (d) true negative if the disease was absent, and a Rightward Bias was not flagged in the SIE scores.

#### 2.5.4. Subsidiary Goals

*Outlying responses.* Concerning the issue of intra-individual variability of performance related to outlying responses, the coefficient of variation (CoV) was used to compute intra-individual variability in the BRIT bisection performance. A common way to reduce the effect of outlying responses is using medians instead of means (the latter being used by the Spreadsheet to automatically compute all scores). This was simply accomplished by replacing the Excel functions accordingly (cf. cells in row 19 and columns E to J in the Spreadsheet). Then, BRIT scores based on medians were used to recompute sensitivity and specificity on the new resulting classification of true/false positives/negatives. Please note that medians were only used to test this specific subsidiary goal, and that our main results (cf. main aim) are based on means.

*One* vs. *two lines.* Concerning the issue of whether a SIE score needs to flag a Rightward Bias in both line lengths (i.e., SIE80 and SIE160), or just one, to indicate the presence of a VHFD, a Fisher exact test was carried out to check for a possible association between the number of lines (one vs. two) returning a Rightward Bias and the number of true/false positives.

*Length Effect.* The LE index is based only on neutral configurations (i.e., plain lines) and represents the difference between bisections in the long and the short line (i.e., LB160—LB80). The Spreadsheet computes the LE score automatically and returns either a “High Bias” (i.e., positive values exceeding the cut-off of 6.7%) or a “Low Bias” (i.e., negative values exceeding the cut-off of −6.5%) flag. A high bias in the LE occurs when the bisection of the long line (LB160) is proportionally more shifted to the right than the bisection of the short line (LB80). Conversely, a low bias in the LE occurs when the bisection of the short line is proportionally more shifted to the right than the bisection of the long line. Since many neglect patients showed a high bias in the LE, one may wonder whether such an index (based on plain lines such as the ones used in the standard bisection task) may be sufficient to detect a VHFD. To this aim, we recomputed sensitivity and specificity based on LE scores instead of SIE scores.

*Leftward Bias in the SIE score.* SIE scores may assume positive or negative values. Negative values mean that the symmetry of illusory effect is shifted to the left (i.e., a patient is more sensitive to the illusion of length expansion on the left than on the right). Left deviations are generally flagged as “Normal” in RBD patients, but may be flagged as a “Leftward Bias” whenever they exceed the (left) cut-off. A Fisher exact test was used to check for a possible association between the presence of a Leftward Bias and false negatives.

## 3. Results

### 3.1. Standard Neuropsychological Assessment of Neglect

Forty-five patients were diagnosed as N+ and 19 as N–. Table 1 reports the demographic and clinical data separately for N+ and N– patients. N+ and N– significantly differed in all tests of the battery for neglect (*p* < 0.001 for all comparisons). The N+ group included 28 patients with ischaemic and 17 with haemorrhagic stroke. The N– group included 16 patients with ischaemic and 3 with haemorrhagic stroke. No significant differences were found between ischaemic and haemorrhagic stroke subgroups in both N+ and N– (*p* > 0.05 for all tests).

### 3.2. Standard Visual Field Assessment

All patients in the final sample underwent reliable visual field examinations with the HFA (see individual outcomes in column “Perimetry outcome (left VHFD)” in Supplementary Table S1). In the N+ group, there were 18 patients with left homonymous hemianopia, 4 with left inferior quadrantanopia, and 1 with left superior quadrantanopia, for a total of 23 N+H+, and 22 N+H–. All patients in the N– group did not have any visual field deficit.

### 3.3. N+H+ and N+H– Groups

Table 1 reports the demographic and clinical data, separately for N+H+ and N+H– patients. Groups were comparable for all demographic variables, and tests of the battery for neglect, except Line cancellation, Line bisection, and Sentence reading (all *p* values < 0.001).

### 3.4. The Brentano Illusion Test (BRIT)

**Figure 1:**
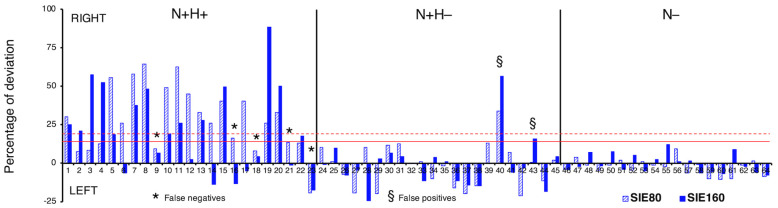
Individual SIE80 and SIE160 scores of the BRIT. Individual left- (negative) and rightward (positive) percentages of deviation (i.e., SIE scores) for the short (80 mm; shaded bars) and long (160 mm; full colour bars) lines as for 23 N+H+ (left panel), 22 N+H– (middle panel), and 19 N– (right panel) patients. The red horizontal lines represent the SIE cut-offs for the short (+19.50; dashed) and long (+14.75; continuous) lines. False negatives are identified by an asterisk, false positives by a section sign.

The average scores for the 5 BRIT indexes are reported in Table 1 separately for N+, N–, N+H+, and N+H–. N+ and N– significantly differed in all BRIT scores (*p* < 0.001), except LB80 and SIE160. N+H+ and N+H– significantly differed only in SIE80 (*p* < 0.001). The SIE scores of 20 patients in the N+ group flagged a Rightward Bias (either in the short or long line, or both; see column “Line(s) to detect VHFD” in Supplementary Table S1), indicating the probable presence of a left VHFD (cf. next section). The SIE scores of 25 patients in the N+ group did not flag any Rightward Bias. Figure 1 shows the individual deviations of SIE scores (i.e., values in columns “SIE80” and “SIE160” in Supplementary Table S1), separately for N+H+, N+H–, and N– patients, along with the cut-offs to flag a Rightward Bias.

### 3.5. Sensitivity and Specificity of the BRIT

**Figure 2:**
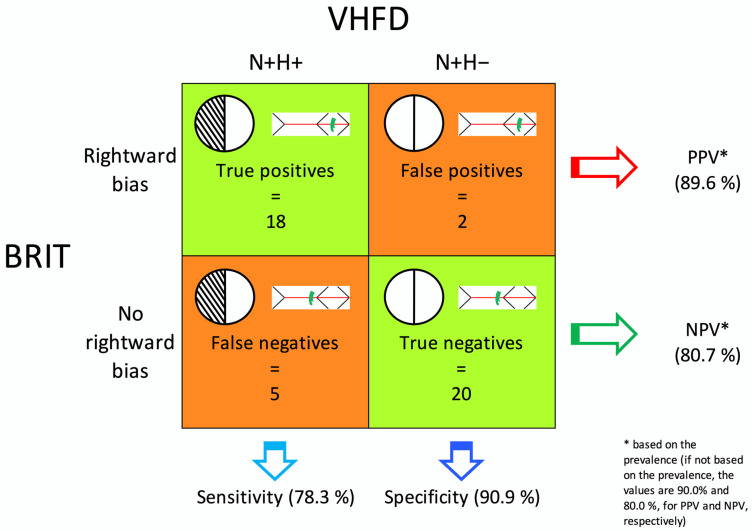
Overview of the accuracy of the BRIT. Number of true/false positive/negative cases classified based on the presence/absence of the VHFD (as assessed by the Humphrey automated static visual field test) and the BRIT outcome (presence/absence of a Rightward Bias in the SIE score). Sensitivity, specificity, PPV, and NPV based on such figures are also reported (cf. Section 3.5).

Neglect patients were classified as follows, based on the presence/absence of a VHFD (as determined by the reference standard) and of a Rightward Bias in the SIE scores. Eighteen patients with a VHFD and with a Rightward Bias were classified as true positives; two without a VHFD and with a Rightward Bias as false positives. Five patients with a VHFD and without Rightward Bias were classified as false negatives; twenty without a VHFD and without a Rightward Bias as true negatives. No false positives were found in the N– group. Supplementary Table S1 shows the individual classification outputs (see column “Classification output”) for the 64 patients. Based on these figures, the sensitivity and specificity of the BRIT were 78.3% (95% CI, 61.4–95.1) and 90.9% (95% CI, 75.2–100.0), respectively. PPV was 89.6% (95% CI, 76.4–100.0) and NPV 80.7% (95% CI, 65.2–96.2). PPV and NPV were computed considering 50% prevalence [43]. Results are summarised in Figure 2. The overall accuracy of the BRIT was 84.4% (this index is reliable since the prevalence of the present set of results is 51% [44]).

As reported in the Original Work, the BRIT cannot tell the type of VHFD, but may potentially detect the presence of a VHFD when a quadrantanopia is present (within the limits of sensitivity and specificity reported in the present study). Indeed, the BRIT detected two cases (inferior quadrantanopias) but failed to detect three (two inferior and one superior). A Fisher exact test showed that there was a significant association (*p* < 0.05) between the type of VHFD and the BRIT capacity to detect it, indicating that hemianopias are more likely detected as true positives than quadrantanopias.

### 3.6. Subsidiary Goals

#### 3.6.1. Outlying Responses

N+ patients in our sample often showed intra-individual variability across trials of the same stimulus condition. On average, N+ showed high variability for all stimulus conditions (CoV > 0.50), except for the long line with the illusion of length expansion to the right (CoV = 0.39), indicating that means may not represent a reliable indicator of performance.

When BRIT scores were recomputed using medians, specificity, sensitivity, PPV, and NPV (and related CIs) returned exactly the same values as computed using means. Supplementary Table S1 also shows BRIT scores based on medians. There was just a change at the individual level (i.e., a change in the classification output of two cases). That is, a false negative—case #18 in Supplementary Table S1—turned into a true positive (the CoV was greater than 1.0 for both line lengths in the neutral configuration), while a true positive—case #22—turned into a false negative (the CoV was 0.45 and 0.60 for the short and long neutral configurations).

#### 3.6.2. One vs. Two Lines

Overall, out of 18 true positives, 4 patients with VHFD (all with hemianopia) were detected by the short line only (i.e., SIE80; see column “Line (s) to detect VHFD” in Supplementary Table S1), 4 patients (3 with hemianopia and 1 with inferior quadrantanopia) by the long line only (i.e., SIE160), and 10 patients (9 with hemianopia, and 1 with inferior quadrantanopia) by both lines. The two false positives were one patient showing a Rightward Bias in the long line, and one showing a Rightward Bias in both lines. However, there was no association between the number of lines and true or false positives (Fisher exact test not significant at *p* < 0.05).

#### 3.6.3. Length Effect

As a group, N+H+ patients showed a high bias in the LE (14.7%; cut-off ≥ 6.7%), indicating that they proportionally bisected the long line more to the right with respect to the short one, but did not significantly differ from N+H– (see Table 1). Consistently with this, most N+H+ patients showed a high bias in the LE at the individual level (see column “LE” in Supplementary Table S1). Sensitivity and specificity recomputed using the LE score instead of the SIE score were 82.6% (95% CI: 67.1–98.1) and 68.2 (95% CI: 48.7–87.6), respectively. Positive predictive value (PPV) was 72.2% (95% CI: 55.1–89.4); negative predictive value (NPV) was 79.7% (95% CI: 61.6–97.8). Therefore, even if a high bias flagged in the LE score detected four out of five cases with a true VHFD that were missed by the SIE scores (cases #9, #16, #18, and #23, see Supplementary Table S1), overall it returned more false positives than the SIE, and hence it cannot be considered a feasible alternative to the SIE for screening VHFD in neglect patients.

#### 3.6.4. Leftward Bias in the SIE Score

Some patients, both in the N– and the N+ groups, showed negative SIE scores (see Supplementary Table S1). Some of these negative scores exceeded the cut-off and were flagged as a Leftward Bias only in N+ patients (7 N+H− and 3 N+H+). Anyway, in the present sample, the presence of such Leftward Biases was not associated with false negatives (Fisher exact test not significant at *p* < 0.05), and did not correspond to any right VHFD.

## 4. Discussion

This study originated from the need to ascertain the presence of possible visual field losses (i.e., left homonymous hemianopia or quadrantanopia) in RBD patients with neglect at the time of neuropsychological assessment, when formal tests such as a computerized visual field are either not possible or not yet available.

In such cases, a practical solution could be the use of an already existing paper-and-pencil test such as the BRIT [23], which is quick and easy to administer to neglect patients for a first screening for possible VHFD. The BRIT represents the most recent work based on the Brentano illusion [24,25,26,27], which reports normative data on healthy adults, but does not provide indications about the accuracy of the test. Therefore, we felt urged to address this issue (as well as others, see below), by designing a study to determine the sensitivity and specificity, as well as the PPV and NPV of this test. If the BRIT is effective, it may conveniently be used as a quick and simple way to screen neglect patients for the presence of VHFD and—in case of a Rightward Bias flagged in the SIE score—to immediately refer them to an orthoptist/ophthalmologist.

One may wonder whether the screening for VHFD on confrontation tests could be performed without any need to resort to the BRIT at all, before referring patients with suspect VHFD to the standard visual field examination. However, screening a VHFD by confrontation tests may not be a feasible alternative (or an additional) test to the BRIT, because many neuropsychologists are not trained, or do not feel confident to perform reliable confrontation tests [21]. In our experience, many colleagues would rather rely on a paper-and-pencil test which easily engages both the patient and the examiner (as is the case while administering the standard battery for neglect).

### 4.1. Sensitivity and Specificity of the BRIT

Our study shows that the BRIT is an effective instrument to screen for VHFD, as it is characterized by high sensitivity and specificity. Additionally, the overall accuracy is high and reliable [44]. We found that the BRIT is excellent to avoid the detection of a deficit in patients with no VHFD (i.e., avoiding false positives), and very good to correctly detect a deficit when it is present in patients who have been diagnosed by the HFA (i.e., true positives). The 18 true positive cases were 16 patients with left homonymous hemianopia and 2 with left inferior quadrantanopia. The five false negative cases were two patients with left homonymous hemianopia, two patients with left inferior quadrantanopia, and one with left superior quadrantanopia (see Supplementary Table S1).

As regards quadrantanopia, and the chance that the BRIT may not detect it (false negatives), the Original Work reported that the BRIT can only detect whether a VHFD is present, but not its type (i.e., hemianopia vs. quadrantanopia), and that quadrantanopias may correspond to either the presence or absence of a Rightward Bias in the SIE score. In the present study, the BRIT succeeded in detecting two cases (inferior quadrantanopia), and failed to detect three (two inferior, one superior). The remaining two false negatives had hemianopia. It seems therefore that quadrantanopias went more easily undetected with respect to hemianopias [21], but a larger sample of patients with quadrantanopia is necessary to confirm this result. Therefore, consistently with the Original Work, we can conclude neither that the BRIT is sensitive to quadrantanopias, nor that it fails to identify patients with quadrantanopia. Given that the prevalence of quadrantanopias following stroke is much lower than that of hemianopias [16,17], any study aiming to address such an issue should use a sample size substantially larger than the one adopted in the present one [28].

As regards false positives, previous studies using the Brentano illusion with neglect patients did not report the frequency of false positives because their focus was not the accuracy of the BRIT ([25,26,27]; also cf. the Original Work). Conversely, we found two false positives (see Supplementary Table S1). One case showed a Rightward Bias in the SIE score in both the short and long lines, while the other in the long line only. Such a low rate of false positives is one of the strengths of the BRIT as a screening test for VHFD.

Based on the HFA outcomes, we did not expect any Rightward Bias in the SIE score in patients without neglect, and indeed there were none (i.e., no false positives). Patients without neglect were tested because we enrolled consecutive RBD patients that were going to be assessed for neglect anyway. To the best of our knowledge, the present work is the first to collect BRIT data in RBD patients without neglect and without any VHFD, nicely complementing the information already available in healthy controls (cf. Original Work), i.e., confirming that it is characterized by a high probability of not returning false positives in individuals without neglect.

**Figure 3:**
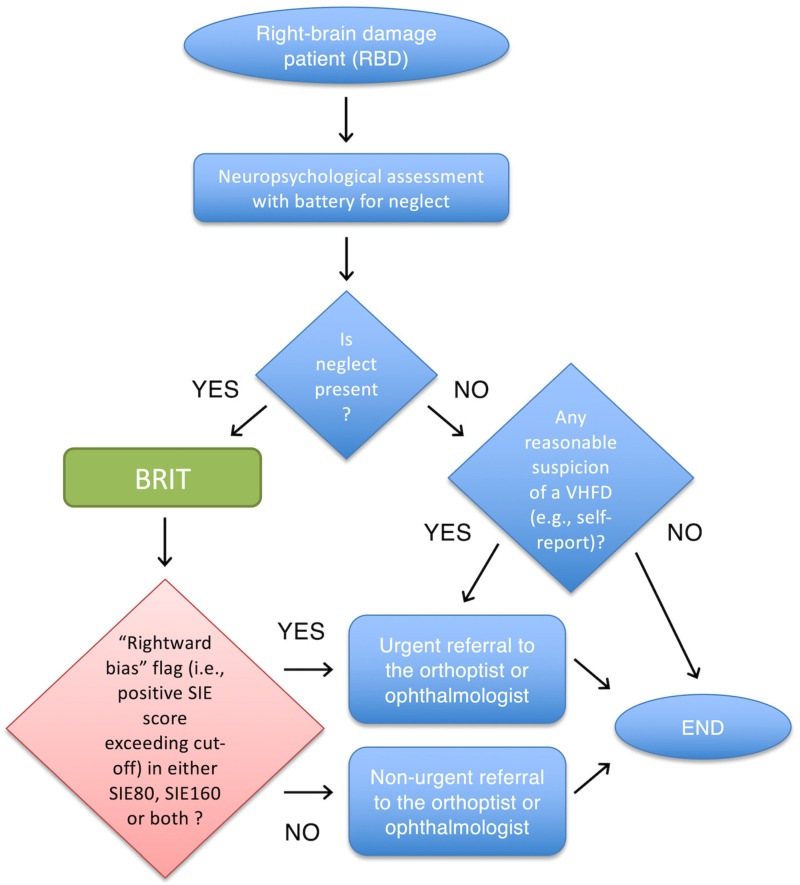
Decision tree chart. Diagram showing the clinical decision-making process to be made about patients admitted to the hospital with a diagnosis of RBD. The terms “urgent” and “non-urgent” are in consideration of both availability of resources and costs (e.g., time and unnecessary clinical examinations). These may differ across different hospitals and their policies. The BRIT outcome can also be used to refine the clinical picture of a patient in the neuropsychological report.

Finally, of greater relevance for the neuropsychological assessment is that the PPV of the BRIT (computed considering the prevalence of VHFD in neglect patients) is high (89.6%). This way, the neuropsychologist knows that when a patient shows a Rightward Bias in the SIE score, an actual visual half-field loss is highly likely, and the patient can be immediately referred for a formal perimetry (cf. Figure 3). Such information is also useful to refine the clinical picture of a patient in the neuropsychological report. The accuracy of a negative BRIT outcome (a “Normal” flag; i.e., NPV) for a single patient is also high, although not as much as the PPV (80.7%). So, when the BRIT does not flag any Rightward Bias in the SIE score, even if it is likely that the patient is correctly screened as not having a VHFD, the neuropsychologist may still consider referring the patient for a non-urgent formal visual field examination.

#### 4.1.1. Big Data

In line with the above-mentioned comments concerning the need of larger samples of neglect patients to address the issue of quadrantanopias, as well as other issues (such as stratifying by relevant demographic/clinical variables), one may consider the recent development of Big Data. Indeed, Big Data are recently becoming more and more important in the medical field, as in many other fields (e.g., [45]). One current trend is the definition of screening and diagnostics protocols using appropriate algorithms [46]. Provided that a standardisation of methodology is guaranteed across different centres collecting data to contribute to large databases, the availability of larger and richer datasets from multicentric studies [47] may represent a valuable source of information to improve sensitivity and specificity of screening and diagnostic tests [46]. Of course, multicentric databases also imply critical issues related to management and access by third-parties (incl. data protection regulations; [48]). This is also very relevant to the neuropsychological field (e.g., making use of well-designed electronic clinical records and sharing them among healthcare institutions; [49]). In fact, although neglect patients are quite frequent among RBD patients [1,4], single-centre studies—such as the present one—unfortunately do not allow the collection of very large sample sizes, which would help refining sensibility and specificity, for instance by adding stratification criteria (e.g., age, gender, time since stroke, neglect severity, etc.). One can anticipate that, as soon as the technical and legal issues still pending are solved, Big Data will revolutionise many fields, including the medical (and neuropsychological) one [50]. Very soon indeed, feeding an algorithm on multimodal (e.g., demographic, behavioural, clinical, neuropsychological, neuroimaging, etc.) data will probably allow a precise diagnosis of most diseases, including neglect and VFD. We hope that the present work may make a contribution in this direction by supporting the adoption of measures that will help disentangling the two.

#### 4.1.2. Implementation Costs and Complexity

We are well aware that healthcare environments may vary substantially across countries, as well as in public vs. private systems, each one characterized by smaller or larger differences in their overall objectives, priorities, and—above all—facilities and availability of resources. Hence, it is very hard to provide figures to help calculate the financial impact of the present protocol, both from a clinical and scientific perspective. However, we can provide information about the resources needed (both human and facilities).

We believe that the adoption of the BRIT in clinical practice can be considered relatively low-cost. In the context of a daily hospital routine, screening patients for VHFD using the BRIT does not require extra dedicated personnel. In fact, the administration and interpretation of the test may be managed by any neuropsychologist already employed in the healthcare institution, while the automated visual field assessment (if needed) can be handled by any orthoptist or ophthalmologist already working in the same environment. As we have reported in the Methods section, the administration of the BRIT should take about 5 min per patient (plus about 10 min for offline scoring). On the other hand, a visual field assessment with the HFA takes about 20–30 min per patient. Hence, the costs (both in terms of expenditure and time) for the healthcare institution depend on the number of patients actually assessed by the neuropsychologist, and possibly referred to the orthoptist/ophthalmologist. The overall accuracy of the BRIT (true positives and negatives) should help optimising referrals to the orthoptist/ophthalmologist, so that they are not too taxing for the healthcare institution.

However, if one intends to replicate the present study (or run a similar one), in addition to the above-mentioned professionals (i.e., neuropsychologist and orthoptist/ophthalmologist), one should also include a senior researcher (responsible for the scientific content and for liaising with the various people involved in the study), and a junior one (e.g., research assistant, PhD student, resident, etc.) who would physically interact with patients, administer and score the BRIT (while being blind to patients’ diagnosis), and take care of data storage and data processing. 

In terms of facilities, in addition to an ophthalmic room, the healthcare institution only needs to be equipped with a suitable testing room. Such a room does not need to be exclusively dedicated to the administration of the BRIT, but can rather be shared by different workers and professionals. The important thing is that it can be set up in a consistent manner across patients to guarantee homogeneous testing conditions. We provide a formalised decision tree chart (cf. Figure 3) to help the clinical decision-making process when screening RBD patients with the BRIT (i.e., to make decisions about whether to refer a patient to the orthoptist/ophthalmologist or not). We believe that the present protocol will help healthcare services with both clinical decision-making (i.e., organisation) and optimising resources (human, financial, and facilities alike).

### 4.2. Subsidiary Goals

During our routine assessment practice with neglect patients, we came across a number of doubts and perplexities about the BRIT (in addition to the issue of the lack of information about the accuracy of the test) that were not addressed in previous studies, such as the impact of a high intra-individual variability across trials (i.e., outlying responses) on the BRIT accuracy, or how to interpret the BRIT outcome when the Spreadsheet flags a Rightward Bias in the SIE score only in a single line (i.e., either long or short), but not both. Other questions were what to do and how to interpret the Length Effect and a Leftward Bias in the SIE score in RBD patients. Such scores are automatically computed by the Spreadsheet, but are not discussed or commented on in the Original Work.

#### 4.2.1. Outlying Responses

Recomputing sensitivity and specificity based on medians (instead of means) did not change our results overall (i.e., we obtained the exact same values), although there were two changes at the individual level (i.e., one false negative turned into a true positive, and one true positive turned into a false negative). This means that, despite the variability of bisection performances may in principle affect the BRIT outcome, its overall impact is indeed minimal and insufficient to alter the test accuracy (that is, the accuracy of the BRIT is not affected by outlying responses). This is a reassuring result that adds to the main sensitivity and specificity results (cf. Section 4.1).

#### 4.2.2. One vs. Two Lines

As regards the question of whether a SIE score needs to flag a Rightward Bias in both line lengths (or whether just one is sufficient) to detect a VHFD, we could not retrieve such information in previous studies [25,26,27]. The few examples presented in the Original Work included only one patient with a VHFD showing a Rightward Bias in the SIE score in both line lengths, but there was no mention that the detection of a VHFD requires a specific criterion. In the present study, the outcome of a Rightward Bias in the SIE scores showed a strong association with the actual presence of a VHFD (i.e., as diagnosed by the reference standard), and there was no association between the BRIT accuracy and the number of lines returning a Rightward Bias. Hence, if a patient shows a Rightward Bias in either line length (or both), the patient is likely to present with a VHFD. Anyway, a formal computation of sensitivity and specificity separately for each line length could not be carried out in the present study, as a larger sample of patients would be needed [28]. In this regard, multicentric studies and large databases would be extremely helpful (cf. Section 4.1.1).

#### 4.2.3. Length Effect

The Length Effect (LE; based on neutral configurations, i.e., plain lines) is the difference between the bisections of the long and the short plain lines. A high LE may seem a good indicator of the presence of a VHFD. In fact, in the N+H+ group most patients showed a High Bias in the LE (cf. Table 1; see also Supplementary Table S1). Therefore, it may seem that the LE detects the presence of a VHFD. However, using this index (instead of the SIE), although sensitivity slightly increased (4 more true positives), specificity dropped (7 more false positives). Given the figures reported above, the LE should be considered as performing worse than the SIE (i.e., the index adopted in the Original Work and the main results of the present study), and therefore does not seem to represent a feasible alternative index. Finally, the fact that high LE scores can also be found in N+H+ patients when they bisect both plain lines on the left half (i.e., negative LB scores) is a result which deserves further investigation.

#### 4.2.4. Leftward Bias in the SIE Score

Some patients showed negative SIE scores, some of which exceeded the cut-off and flagged a Leftward Bias in one or both line lengths. Our analysis showed that the presence of a Leftward Bias in the SIE scores was not associated with false negatives (but studies with larger samples are needed, since this result is based on a few false negatives showing a Leftward Bias). A Leftward Bias (exactly like a Rightward Bias) depends on the algebraic relationship between the positions of the bisections across the three configurations (neutral, illusion of length expansion on the left, and illusion of length expansion on the right). It is worth noticing that a Leftward Bias is not necessarily due to bisecting the “illusion of length expansion on the left” on the left half of the line, while bisecting the other two configurations on the right half. Rather, it may be simply due to bisecting the “illusion of length expansion on the left” more to the left than the other two configurations. Anyway, the presence of a Leftward Bias in RBD patients does not correspond to a right VHFD. Therefore, it would be interesting to investigate how the Leftward Bias performs in patients with a left-brain damage and a right unilateral spatial neglect. However, neglect in left-brain damage patients is much less frequent than in RBD patients [1]. Again, multicentric studies and large databases would probably be needed to investigate such a question (cf. Section 4.1.1).

### 4.3. Practical Suggestions

Finally, we would like to provide some practical tips for printing and scoring the test. Before printing the “BRIT_Test.pdf” file, we recommend checking (and accordingly adjusting) the percentage of magnification of the actual print in the printer settings. Otherwise, the test may not correctly display the red lines length (80 and 160 mm) and thickness. For the scoring procedure, one may conveniently enter the measured values directly into the Spreadsheet (making sure to create a separate sheet for each patient), rather than writing them down in the printed file “BRIT Scoresheet.pdf”, and then entering them into the Spreadsheet as a second step. This is because the cut-offs in the Scoresheet seem to be inconsistent with both the Spreadsheet and the paper’s content (cf. Original Work).

## 5. Conclusions

The aim of the present study was to determine the sensitivity and specificity of the BRIT as a screening test for VHFD in RBD patients with unilateral spatial neglect. The BRIT showed high sensitivity and specificity, indicating that it is effective for screening visual deficits in neglect patients who are truly affected by a VHFD (as ascertained by the reference standard), and avoiding false positives in neglect patients who are truly not affected by a VHFD. In addition, at the individual level, when the BRIT flags a Rightward Bias in the SIE scores, it is highly likely (PPV) that the neglect patient presents with a VHFD, and the patient should be urgently referred for a formal perimetry. Conversely, when the BRIT does not flag a Rightward Bias in the SIE scores, it is likely (NPV) that the neglect patient does not present any VHFD, but the option of (non-urgently) referring the patient for a formal perimetry nonetheless (depending on a given healthcare institution’s resources/policies) may still be considered. Therefore, the BRIT represents a useful tool for the neuropsychologist to screen for VHFD when assessing neglect patients. Its administration is quick and easy, and it may add information relevant for: (a) refining the clinical picture of a patient in the neuropsychological report; (b) urgently referring a patient for a formal perimetry.

## Figures and Tables

**Table 1:** Demographic and clinical data by group, incl. battery for neglect and BRIT scores. Data are reported separately for N+ (irrespective of VHFD) and N– groups, and for neglect patient groups with or without a VHFD (N+H+ and N+H–, respectively). Positive values in the cancellation and line bisection tasks of the battery for neglect indicate performance shifted towards the right hemispace. [Please see Section 2.5.2 for an explanation of the following indexes and scores of the BRIT]. Positive values in LB80 and LB160 (Line Bisection in the neutral configuration for the short and long line, respectively), and SIE80 and SIE160 (Symmetry of the Illusory Effect for the short and long line) represent deviations to the right. Negative values in SIE80 and SIE160 represent deviations to the left. Positive LE values (Length Effect, approx. [LB160–LB80], after adjusting for age) indicate that bisections of the long line are proportionally more shifted to the right than bisections of the short line. BRIT cut-offs for the positive values are reported. The SIE scores are reported both in millimeters and as percentages (cf. Spreadsheet). Values in parentheses represent SD. * A cut-off ≤ 5 correct sentences corresponds to a cut-off ≥1 sentences read incompletely on the left side (cf. Section 2.2).

		N+	N–	Comparison	N+H+	N+H–	Comparison
**Demographics**							
N		45	19		23	22	
Sex		26 M; 19 F	9 M; 10 F	n.s.	14 M; 9 F	12 M; 10 F	n.s.
Age (years)		60.9 (13.1)	64.9 (14.1)	n.s.	57.4 (13.5)	64.5 (12.0)	n.s.
Education (years)		12.0 (4.7)	11.9 (4.8)	n.s.	13.1 (4.3)	10.8 (5.0)	n.s.
Time since stroke (days)		80.0 (73.5)	44.8 (28.7)	n.s.	94.9 (81.3)	64.5 (62.4)	n.s.
N Ischaemic stroke		28	16	n.s.	14	14	n.s.
N Haemorrhagic stroke		17	3	n.s.	9	8	n.s.
							
**Battery for neglect (at admittance)**	**Cut-offs**						
Letter cancellation (spatial bias)	≥4	19.0 (14.4)	1.0 (1.9)	<0.001	20.3 (12.1)	17.8 (16.6)	n.s.
Star cancellation (spatial bias)	≥3	10.3 (9.2)	0.4 (0.9)	<0.001	10.7 (8.8)	10.0 (9.9)	n.s.
Line cancellation (spatial bias)	≥2	2.7 (3.5)	0.0 (0.0)	<0.001	4.4 (3.8)	1.0 (2.1)	<0.001
Apples test accuracy (N targets)	≤44	22.4 (14.8)	46.0 (10.4)	<0.001	19.6 (14.2)	25.2 (15.2)	n.s.
Apples test space-centered (asymmetry score)	≥3	9.1 (6.6)	0.1 (0.9)	<0.001	9.3 (6.1)	8.8 (7.3)	n.s.
Apples test object-centered (asymmetry score)	≥2	8.5 (10.8)	0.4 (1.3)	<0.001	11.0 (11.3)	6.1 (10.1)	n.s.
Line bisection 200 mm (% of shift from the midpoint)	>6.73	21.5 (24.3)	2.3 (3.5)	<0.001	33.0 (28.2)	9.4 (10.6)	<0.001
Wundt-Jastrow illusion (N unexpected responses)	≥2	8.8 (7.9)	0.2 (0.5)	<0.001	11.5 (7.8)	6.0 (7.2)	n.s.
Sentence reading (N correct sentences)	≤5 *	3.5 (2.6)	6.0 (0.0)	<0.001	2.2 (2.6)	4.8 (1.8)	<0.001
Personal neglect (asymmetry score)	≥2	1.8 (2.0)	0.2 (0.7)	<0.001	1.5 (1.8)	2.2 (2.2)	n.s.
Room description (asymmetry score)	≥1	1.5 (1.2)	0.1 (0.2)	<0.001	1.4 (1.2)	1.5 (1.2)	n.s.
							
**BRIT**	**Cut-offs**						
LB80 (%)	≥8.50	5.4 (16.9)	1.1 (2.4)	n.s.	9.7 (21.2)	1.0 (9.2)	n.s.
LB160 (%)	≥12.25	15.0 (21.5)	1.4 (3.8)	<0.001	24.5 (26.2)	5.1 (7.2)	n.s.
LE (%)	≥6.7	9.6 (11.3)	0.4 (3.9)	<0.001	14.7 (13.1)	4.3 (5.5)	n.s.
SIE80 (mm)	≥7.8	5.5 (9.4)	−1.0 (2.1)	<0.001	11.5 (8.5)	−0.7 (5.6)	<0.001
SIE80 (%)	≥19.5	13.9 (23.5)	−2.5 (5.2)		28.7 (21.2)	−1.6 (14.0)	
SIE160 (mm)	≥11.8	8.6 (19.9)	−0.1 (4.8)	n.s.	17.2 (21.7)	−0.4 (12.8)	n.s.
SIE160 (%)	≥14.75	10.8 (24.8)	−0.2 (6.0)		21.6 (27.2)	−0.5 (16.0)	

## Data Availability

The data presented in this study are available on request from the corresponding author. The data are not publicly available due to privacy policies.

## Supplementary Materials

The following supporting information can be downloaded at: https://www.mdpi.com/article/10.3390/brainsci13060937/s1, Table S1. Demographic and clinical data and BRIT scores by individuals.

## Abbreviations

BRIT	Brentano Illusion Test
CI	confidence interval
CoV	coefficient of variation
HFA	Humphrey Field Analyzer
LB	line bisection
LE	length effect
N+	patients with neglect
N–	patients without neglect
N+H+	neglect patients with a left visual hemi-field deficit
N+H–	neglect patients without a left visual hemi-field deficit
NPV	negative predictive value
PPV	positive predictive value
RBD	right-brain damage
SIE	Symmetry of the Illusory Effect
VFD	visual field deficits
VHFD	visual hemi-field deficits
