# Supplementary material for: Sensitivity and Specificity of the Brentano Illusion Test in the Detection of Visual Hemi-Field Deficits in Patients with Unilateral Spatial Neglect
Source: Brain Sci. 2023 Jun 9;13(6):937. doi: 10.3390/brainsci13060937 (PMC10296626; doi:10.3390/brainsci13060937)
Supplement: Supplementary file 1 [file brainsci-13-00937-s001.zip › brainsci-2398862-supplementary.pdf]

Supplementary Table S1. Demographic and clinical data and BRIT scores by individuals. Clinical data include time since stroke, aetiology, and the outcome of the visual field examination based on static automatic perimetry ("hemianopia", inferior quadrantanopia – "inf Q", or superior quadrantanopia – "sup Q"). As regards the BRIT, the following data are reported: *classification output* (true or false positives or negatives), *line(s) to detect VHFD* (short, long, or both) flagging a Rightward bias, and the scores of the 5 *BRIT indexes* (LB80, LB160, LE, SIE80, and SIE160). Positive values in LB80 and LB160 (Line Bisection in the neutral configuration for the short and long line, respectively), and SIE80 and SIE160 (Symmetry of the Illusory Effect for the short and long line), represent deviations to the right. Negative values in LB80, LB160, SIE80 and SIE160 represent deviations to the left. Positive LE values (Length Effect, approx. [LB160–LB80], after adjusting for age) indicate that bisections of the long line are proportionally more shifted to the right than bisections of the short line. Negative LE values indicate that bisections of the long line are proportionally more shifted to the left than bisections of the short line. BRIT data are reported separately for the computations based on means (main results) and based on medians (outlying responses; cf. sections 2.5.4 and 3.6.1). Values in bold exceed the cut-offs.

								**Based on means (main results)**							**Based on medians (outlying responses)**						
**Group**	**Patient ID**	**Sex**	**Age (years)**	**Education (years)**	**Time since stroke (days)**	**Aetiology**	**Perimetry outcome (left VHFD)**	**Classification output**	**Line(s) to detect VHFD**	**LB 80 %**	**LB 160 %**	**LE %**	**SIE 80 %**	**SIE 160 %**	**Classification output**	**Line(s) to detect VHFD**	**LB 80 %**	**LB 160 %**	**LE %**	**SIE 80 %**	**SIE 160 %**
N+H+	1	M	38	13	42	haemorrage	hemianopia	true pos.	both	**32.5**	**53.5**	**20.4**	**30.0**	**25.2**	true pos.	both	**37.5**	**41.0**	2.9	**22.5**	**53.5**
N+H+	2	M	33	8	44	haemorrage	hemianopia	true pos.	long	**-12.0**	6.9	**18.2**	7.5	**21.0**	true pos.	long	**-12.5**	11.1	**23.0**	12.5	**20.5**
N+H+	3	F	42	18	76	haemorrage	hemianopia	true pos.	long	**12.8**	**31.6**	**18.4**	8.5	**57.6**	true pos.	long	**12.5**	**32.1**	**19.1**	-7.5	**53.2**
N+H+	4	F	79	18	102	haemorrage	hemianopia	true pos.	long	**-8.5**	-1.8	**7.3**	12.5	**52.2**	true pos.	long	**-10.0**	-4.8	5.8	17.5	**67.9**
N+H+	5	F	66	8	115	haemorrage	hemianopia	true pos.	both	**25.0**	**48.1**	**23.2**	**55.5**	**18.7**	true pos.	short	**20.0**	**56.1**	**36.2**	**65.0**	5.4
N+H+	6	M	54	13	132	haemorrage	hemianopia	true pos.	short	**32.0**	**56.4**	**24.2**	**26.0**	-6.2	true pos.	short	**30.0**	**56.7**	**26.4**	**30.0**	-6.2
N+H+	7	F	50	18	172	haemorrage	hemianopia	true pos.	both	**-16.0**	-0.9	**14.7**	**58.0**	**37.7**	true pos.	both	-7.5	-0.7	6.5	**62.5**	**68.4**
N+H+	8	F	48	13	70	haemorrage	inf Q	true pos.	both	**15.5**	**27.3**	**11.4**	**64.5**	**48.1**	true pos.	both	**13.8**	**32.5**	**18.4**	**68.8**	**35.7**
N+H+	9	M	52	18	71	haemorrage	inf Q	false neg.	-	4.5	**17.4**	**12.6**	9.3	6.6	false neg.	-	6.3	**14.3**	**7.7**	7.5	-0.2
N+H+	10	M	66	13	22	ischaemia	hemianopia	true pos.	both	**16.6**	**49.1**	**32.6**	**49.1**	**19.1**	true pos.	both	**23.4**	**51.5**	**28.2**	**37.7**	**21.4**
N+H+	11	M	55	8	26	ischaemia	hemianopia	true pos.	both	**-12.3**	**13.7**	**25.8**	**62.5**	**26.2**	true pos.	short	**-15.0**	**12.9**	**27.7**	**60.0**	7.7
N+H+	12	F	79	5	35	ischaemia	hemianopia	true pos.	short	**47.5**	**61.6**	**14.8**	**45.0**	2.4	true pos.	short	**55.0**	**61.4**	**7.0**	**37.5**	4.4
N+H+	13	M	67	13	37	ischaemia	hemianopia	true pos.	both	**-13.8**	11.6	**25.4**	**33.0**	**28.0**	true pos.	both	**-13.8**	11.7	**25.5**	**26.3**	**24.9**
N+H+	14	M	69	5	47	ischaemia	hemianopia	true pos.	short	**-17.5**	**18.1**	**35.8**	**26.0**	**-13.8**	true pos.	short	**-20.0**	**18.4**	**38.6**	**35.0**	**-16.5**
N+H+	15	M	77	18	86	ischaemia	hemianopia	true pos.	both	**10.8**	**22.6**	**12.4**	**40.5**	**49.4**	true pos.	both	**15.0**	**19.1**	4.6	**31.3**	**61.3**
N+H+	16	F	75	13	87	ischaemia	hemianopia	false neg.	-	**55.0**	**72.5**	**17.9**	16.5	**-13.4**	false neg.	-	**70.0**	**74.3**	4.7	-15.0	-11.7
N+H+	17	M	49	8	115	ischaemia	hemianopia	true pos.	short	**36.5**	**60.6**	**23.8**	**40.5**	-5.0	true pos.	short	**42.5**	**63.1**	**20.3**	**32.5**	**-14.2**
N+H+	18	M	61	13	161	ischaemia	hemianopia	false neg.	**-**	-1.0	5.8	**6.8**	8.0	4.5	true pos.	short	-7.5	6.3	**13.8**	**20.0**	3.5
N+H+	19	M	53	13	226	ischaemia	hemianopia	true pos.	both	**-9.5**	**-36.4**	**-27.2**	**26.3**	**88.5**	true pos.	both	-6.3	**-35.2**	**-29.2**	**20.0**	**96.2**
N+H+	20	M	63	18	381	ischaemia	hemianopia	true pos.	both	**26.5**	**20.5**	-6.0	**33.0**	**50.1**	true pos.	both	**10.0**	**23.7**	**13.7**	**67.5**	**43.8**
N+H+	21	M	58	18	40	ischaemia	inf Q	false neg.	-	-5.0	1.6	6.5	13.5	-1.3	false neg.	-	-5.0	-1.0	3.9	12.5	4.3
N+H+	22	F	41	13	52	ischaemia	inf Q	true pos.	long	0.3	5.8	5.0	13.0	**17.7**	false neg.	**-**	0.0	2.1	1.6	5.0	5.0
N+H+	23	F	44	16	43	ischaemia	sup Q	false neg.	-	3.5	**17.3**	**13.3**	**-19.0**	**-17.5**	false neg.	-	5.0	**15.2**	**9.7**	**-22.5**	**-22.9**
N+H-	24	F	64	11	17	haemorrage	none	true neg.	-	-3.0	-0.3	2.7	10.5	-0.8	true neg.	-	-3.8	1.2	5.0	16.3	-6.7
N+H-	25	M	54	18	27	haemorrage	none	true neg.	-	**-10.0**	-4.3	5.4	1.3	10.1	true neg.	-	**-12.5**	-4.0	**8.3**	5.0	10.7
N+H-	26	M	85	8	35	haemorrage	none	true neg.	-	5.8	6.8	2.0	-7.0	-7.9	true neg.	-	5.0	6.4	2.4	-8.8	-7.0

N+H-	27	M	77	18	36	haemorrage	none	true neg.	-	8.0	4.1	-3.4	**-19.3**	-4.5	true neg.	-	**10.0**	4.7	-4.8	**-18.8**	-2.4
N+H-	28	M	49	8	61	haemorrage	none	true neg.	-	**-22.8**	-6.0	**16.4**	10.5	**-24.3**	true neg.	-	**-20.0**	-5.6	**14.0**	8.8	**-28.0**
N+H-	29	M	73	11	77	haemorrage	none	true neg.	-	5.5	2.5	-2.6	**-19.5**	3.1	true neg.	-	2.5	5.0	2.9	-15.0	-3.2
N+H-	30	F	67	17	85	haemorrage	none	true neg.	-	-4.3	-5.0	-0.6	11.8	6.5	true neg.	-	-3.8	2.3	6.1	11.3	-6.8
N+H-	31	M	76	8	110	haemorrage	none	true neg.	-	**11.5**	**15.9**	4.9	12.5	4.1	true neg.	-	**12.5**	**16.6**	4.6	15.0	3.6
N+H-	32	M	57	8	22	ischaemia	none	true neg.	-	**10.5**	8.2	-2.5	-0.3	-0.1	true neg.	-	**10.0**	9.0	-1.1	-3.8	-3.3
N+H-	33	M	61	8	27	ischaemia	none	true neg.	-	4.5	11.6	**7.1**	1.0	-11.2	true neg.	-	5.0	**13.2**	**8.2**	7.5	**-14.7**
N+H-	34	F	78	5	28	ischaemia	none	true neg.	-	**15.5**	**13.9**	-1.1	-9.8	4.0	true neg.	-	**13.8**	9.6	-3.6	-11.3	14.1
N+H-	35	F	52	8	29	ischaemia	none	true neg.	-	-4.0	0.5	4.2	-1.8	1.3	true neg.	-	-5.0	-0.1	4.6	-2.5	2.9
N+H-	36	M	74	6	31	ischaemia	none	true neg.	-	3.0	11.1	**8.5**	-15.8	-11.6	true neg.	-	3.8	10.6	**7.2**	**-18.8**	-10.7
N+H-	37	F	62	5	33	ischaemia	none	true neg.	-	**10.0**	**14.8**	4.8	**-19.8**	**-14.3**	true neg.	-	**10.0**	**14.5**	4.4	**-18.8**	-12.1
N+H-	38	F	81	5	34	ischaemia	none	true neg.	-	**10.3**	9.0	-0.5	-14.5	**-14.5**	true neg.	-	7.5	8.8	2.0	-11.3	**-17.8**
N+H-	39	F	76	13	38	ischaemia	none	true neg.	-	**-12.5**	1.3	**14.3**	13.3	-0.4	true neg.	-	**-13.8**	0.5	**14.7**	16.3	4.7
N+H-	40	F	69	5	45	ischaemia	none	false pos.	both	-2.8	-1.2	1.7	**33.8**	**56.7**	false pos.	long	0.0	-0.3	-0.1	8.8	**68.4**
N+H-	41	M	49	18	51	ischaemia	none	true neg.	-	-5.3	3.0	**7.9**	7.0	-6.0	true neg.	-	-5.0	3.8	**8.4**	7.5	-6.8
N+H-	42	F	46	8	91	ischaemia	none	true neg.	-	-1.0	12.2	**12.8**	**-21.0**	-3.0	true neg.	-	-5.0	**13.2**	**17.8**	-10.0	-0.7
N+H-	43	M	57	18	112	ischaemia	none	false pos.	long	-2.3	-1.0	1.1	0.2	**16.0**	false pos.	long	-2.5	-1.0	1.4	0.0	**20.5**
N+H-	44	F	64	18	126	ischaemia	none	true neg.	-	8.3	**16.8**	**8.6**	-11.3	**-18.1**	true neg.	-	5.0	**16.2**	**11.2**	-2.5	**-16.1**
N+H-	45	M	48	13	303	ischaemia	none	true neg.	-	-4.0	-1.7	1.9	2.0	4.5	true neg.	-	-2.5	-2.5	-0.3	-2.5	6.3
N-	46	M	75	8	36	haemorrage	none	true neg.	-	-1.0	-1.9	-0.4	-3.8	-4.0	true neg.	-	0.0	-3.2	-2.8	0.0	0.8
N-	47	F	45	8	42	haemorrage	none	true neg.	-	0.3	2.4	1.7	4.0	-2.0	true neg.	-	0.0	3.2	2.8	3.8	-3.3
N-	48	M	60	18	83	haemorrage	none	true neg.	-	1.5	-4.6	-6.2	-1.0	7.1	true neg.	-	2.5	-4.9	**-7.4**	-2.5	7.1
N-	49	M	31	18	19	ischaemia	none	true neg.	-	2.3	0.1	-2.9	-4.5	-1.7	true neg.	-	2.5	1.2	-2.0	-3.8	-5.2
N-	50	F	87	13	19	ischaemia	none	true neg.	-	2.8	-1.9	-3.5	-1.3	7.5	true neg.	-	2.5	-1.4	-2.8	-1.3	5.8
N-	51	F	71	5	24	ischaemia	none	true neg.	-	0.0	3.5	3.8	2.3	-4.0	true neg.	-	0.0	3.3	3.6	1.3	-3.7
N-	52	M	70	5	25	ischaemia	none	true neg.	-	-1.0	-0.2	1.0	-4.3	5.4	true neg.	-	-2.5	-0.4	2.3	0.0	4.8
N-	53	F	40	12	26	ischaemia	none	true neg.	-	3.3	8.9	5.2	1.3	-4.9	true neg.	-	3.8	8.4	4.2	0.0	-3.9
N-	54	M	72	18	29	ischaemia	none	true neg.	-	2.0	4.2	2.5	-1.3	2.7	true neg.	-	2.5	4.4	2.3	-3.8	1.5
N-	55	F	67	13	30	ischaemia	none	true neg.	-	2.0	-5.6	**-7.5**	-2.3	12.3	true neg.	-	2.5	-4.6	**-7.0**	-3.8	11.3
N-	56	M	60	16	31	ischaemia	none	true neg.	-	-0.5	-0.2	0.2	9.3	1.1	true neg.	-	0.0	-1.1	-1.2	10.0	2.7
N-	57	F	73	8	31	ischaemia	none	true neg.	-	-0.8	-0.6	0.5	-6.3	1.7	true neg.	-	-2.5	-0.6	2.2	-2.5	0.4
N-	58	M	65	18	36	ischaemia	none	true neg.	-	1.8	6.3	4.6	-0.8	-6.2	true neg.	-	1.3	6.1	5.0	3.8	-6.0
N-	59	M	71	18	38	ischaemia	none	true neg.	-	-1.0	5.2	6.4	-9.8	-5.3	true neg.	-	-2.5	4.5	**7.3**	-6.3	-5.6
N-	60	M	61	13	41	ischaemia	none	true neg.	-	7.8	6.1	-1.7	-10.5	-6.9	true neg.	-	7.5	6.3	-1.2	-7.5	-5.3
N-	61	F	79	13	63	ischaemia	none	true neg.	-	4.8	-0.1	-4.2	-10.0	8.9	true neg.	-	5.0	-1.1	-5.5	-12.5	9.2
N-	62	F	75	5	72	ischaemia	none	true neg.	-	-2.0	-0.9	1.6	-1.3	-1.9	true neg.	-	-1.3	-1.4	0.3	-2.5	-0.4
N-	63	F	75	9	73	ischaemia	none	true neg.	-	-0.8	2.4	3.5	1.5	-5.8	true neg.	-	-2.5	1.7	4.7	3.8	-1.6
N-	64	F	56	8	134	ischaemia	none	true neg.	-	-0.3	3.7	3.8	-8.7	-7.4	true neg.	-	1.3	4.1	2.6	-12.5	**-15.9**
